# Endometrial Cytology During the Different Phases of the Estrous Cycle in Jennies: New Evidences

**DOI:** 10.3390/ani10061062

**Published:** 2020-06-19

**Authors:** Marco Quartuccio, Santo Cristarella, Pietro Medica, Esterina Fazio, Giuseppe Mazzullo, Claudia Rifici, Luigi Liotta, Katiuska Satué

**Affiliations:** 1 Department of Veterinary Sciences, Veterinary Physiology Unit, Polo Universitario Annunziata, Messina University, 98168 Messina, Italy; mquartuccio@unime.it (M.Q.); scristarella@unime.it (S.C.); pmedica@unime.it (P.M.); fazio@unime.it (E.F.); giuseppe.mazzullo@unime.it (G.M.); claudiarifici35@gmail.com (C.R.); luigi.liotta@unime.it (L.L.); 2 Department of Animal Medicine and Surgery, Faculty of Veterinary Medicine, CEU-Cardenal Herrera University, 46115 Valencia, Spain

**Keywords:** cytobrush, endometrial cytology, estrous cycle, jennies

## Abstract

**Simple Summary:**

This study provides new evidences of the physiological changes of endometrial epithelial cells in cycling jennies during the estrus cycle. The number and morphology of endometrial cells significantly change during the estrous cycle. Endometrial epithelial cell exfoliation is significantly more abundant in estrus than diestrus. The morphological pattern of endometrial epithelial cells shows columnar morphology during estrus, becoming more cuboidal during diestrus. Likewise, degenerative changes observed in later diestrus compared with normal morphology of endometrial epithelial cells in estrus are shown. Scarce segmented neutrophils and erythrocytes are a common response during estrus. The cytobrush (CB) technique represents a suitable method for the endometrial evaluation, taking into account cytopathological purposes in cycling jennies.

**Abstract:**

Since in the mare and other animal species such as bitches and cats, the endometrial cell pattern varies depending on the phase of the estrous cycle, the aim of this study was to describe and quantify the endometrial cytological (EC) findings in cycling jennies. EC of eight nonpregnant jennies by cytobrush (CB) at diestrus (day 1 and day 14) and estrous (day 21) were evaluated. All slides were stained with Wright´s stain and microscopically examined at both 400× and 1000× magnification. Seven high-power fields (400×) were assessed in each smear and the endometrial epithelial cells and neutrophils (PMNs) were counted. Endometrial epithelial cells were classified as intact, distorted or fragmented and, on the basis of the presence of dense groups, in monolayer or single clusters. Cytoplasmic characteristics, such as vacuolation or streaming and size, form, position of nuclear characteristics, including karyorrhexis, were recorded. Background aspect, as clear, proteinaceous, or debris, was also considered. In general, sampling by CB provided a yield of cells and clumped endometrial epithelial cells in many smears, being more abundant in estrus than early and late diestrus. Individual endometrial epithelial cells, during estrous, presented a columnar morphology, ciliated or not ciliated and basal nuclei. During diestrus phase, endometrial epithelial cells presented a more cuboidal ciliated or not ciliated morphology. Moderate amount of proteinacious material and red blood cells (RBC) was also observed. Non variation in the percentage of PMNs during diestrus was obtained, but lower and segmented PMNs in CB smears were shown in estrous. This study provides new insights on the physiological changes of endometrial epithelial cells in cycling jennies during the estrus cycle. The CB technique represents a suitable and adequate method for endometrial evaluation, taking into account cytological and/or cytopathological purposes also in jennies.

## 1. Introduction

Uterine integrity is directly related to fertility, so that its estimation is vital during the reproductive evaluation. In the mare, endometrial cytology (EC) is commonly used to evaluate the origin of infertility (acute or chronic endometritis), to provide an immediate diagnosis for clinical management, monitoring the response to treatment of uterine inflammation [1,2,3,4] and to determine the ovulation timing. Among the advantages of cytological examination are the ability to receive results quickly after specimen collection as well as being relatively cheap and easy to perform.

Different methods to collect the endometrial samples, as double guarded cotton swab (DGS), Knudsen catheter, uterine cytobrush (CB) or low-volume uterine (LVL) flush as well as endometrial biopsy, can be used [5,6,7]. Several studies have examined the sensitivity and specificity of these methods for the detection of inflammation and/or endometrial infection in mares [8,9,10]. These techniques returned similar results in jennies and were useful for evaluating the endometrial environment [11]. Although each method has a diagnostic value, their usefulness increases when they are used together [12].

CB is considered superior to the other methods for cytological sampling, since it is easier, more consistent and produces samples with higher cellularity than the other techniques, although a gentle preparation of smear is mandatory to reduce cells distortion [9,10,13]. Although the CB method allows collecting of superficial and deep cells of endometrium up to glandular cells [9], fragmentation and contamination with proteinaceous material have been frequently produced by its invasiveness [12]. However, the CB technique is preferentially used to detect subclinical endometritis in the practice for its inexpensiveness and safeness [8,14,15]. Table 1 shows the cellular types that are usually present on cytological analysis on mares, and that they can be applied to the donkey.

Endometrial epithelial cells are typically columnar, with or without cilia, and they can be observed as individual cells or part of groups, sheets, or clumps of cells [21]. In normal mares, the stage of the reproductive cycle may also affect the numbers and cellular morphologic characteristics [22]. Knudsen [23] showed that endometrial epithelial cells were generally found in orderly groups during diestrus, while Couto and Hughes [21] reported a subjective increase in the number of ciliated endometrial epithelial cells. In addition, proportionately more free endometrial epithelial cells during estrus, and more endometrial epithelial cell rafts during diestrus have been found [22,23].

Donkeys have been gaining in importance as companion, pack and draft animals in some countries; the artificial insemination protocols followed with these animals are usually those designed for mares, with disappointing results [24,25].

Although the jenny is very similar in many reproductive aspects to the mare [26], asinine female reproductive tract reveals several peculiarities compared to the mare. Indeed, jenny has all the vulva under the pelvis floor and higher vaginal vestibule inclination than mare [17,18,19]. Furthermore, jennies have a relatively longer cervix with more tortuous folds [18,27,28] that protrudes into the vagina making artificial insemination more difficult and the ability to conceive after insemination poorer than in mares [11,24,25].

These differences in the anatomy and physiology of the mare’s and jenny’s reproductive tract may predispose the jenny to endometrial inflammation. The conformation of the cervix [18,29] and the lower accumulation of fluid in the endometrium during estrus [19] favor the development of post-insemination endometritis, since they have different uterine cleaning mechanisms than the mare. Research on the endometrial environment in the donkey is scarce.

However, although quantitative changes of inflammatory cells, in response to endometritis [27] and insemination [11,30,31], were observed, morphological characteristics of endometrial epithelial cells in cycling jennies have not been analyzed. The objective of this study was to describe the quantitative and qualitative characteristics of cellularity present in the endometrial lumen during estrus and diestrus in healthy cycling jennies and the relation with age and the number of births.

## 2. Materials and Methods

### 2.1. Animals

All methods and procedures used in this study followed the guidelines of the Italian law (D.L. 04/3/2014 n. 26) and EU directive (2010/63/EU) on the protection of animals used for scientific purposes and were carried out by the Center for Animal Reproduction and Assisted Insemination of the University of Messina. This study did not required approval from authorities or the organisations ethics committees. The study was performed between March and April 2019 at the Sicilian Institute of Horse Breeding. Eight Ragusana jennies, aged 4–16 years, weighing 325 ± 25 kg, were housed in paddocks, with natural photoperiod and fed with hay and a feed-based supplement twice a day. Jennies were considered free of pathologies (infertility, uterine fluid or previous endometritis) and at least 3 ultrasound examinations confirmed the existence of regular cycles. In previous years, the jennies were submitted to the natural breeding, and therefore all animals presented eutocic delivery and all foals suckled until weaning.

### 2.2. Samples Collection

**Figure 1:**
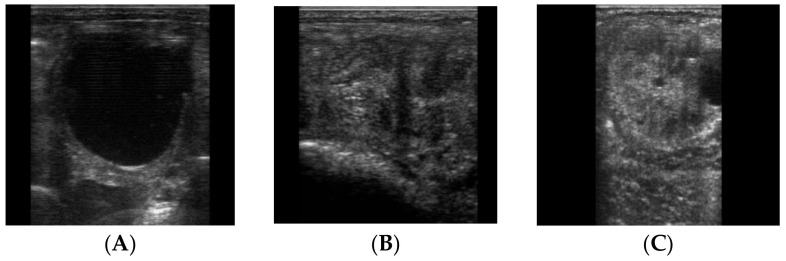
Ecography of the jenny’s ovary. (**A**) Estrus. Dominant follicle (>35 mm in diameter); (**B**) Estrus. Uterine edema, (**C**) Diestrus. Corpus luteum (post-ovulation).

All jennies were submitted to a complete gynecological evaluation, which included a vaginoscopy, rectal exploration and ultrasound of the reproductive tract, to evaluate the physiological state of genitalia, the determination of the phase of estrous cycle and to exclude pregnancy, using a portable ultrasound scanner (Aquila-Esaote-PieMedical, Genova, Italy), equipped with endocavitary linear multifrequency probe (6–8 MHz). The jennies were subjected to the evaluation during two estrous cycles. In the first estrous cycle, they were monitored by ultrasound every two days, and it served both to discard any ongoing reproductive pathology and to synchronize estrus cycles. To monitor the estrous cycle, the size and number of follicles in each ovary were considered, until a dominant follicle (>35 mm in diameter) and detectable uterine edema were identified (Figure 1A,B). After a follicle of >35 mm in diameter was detected, the ovaries of jennies were scanned daily until ovulation that was induced using with 1500 I.U/subject intravenously of human chorionic gonadotropin (Corulon^®^, Intervet, Italia S.r.l.) to synchronize the jennie´s cycles. 24–48 h after the hCG administration, all jennies ovulated (Figure 1C). In the second estrous cycle, the CB collections were performed the day after ovulation (D-1) and at days 14 and 21 (D-14 and D-21) when uterine edema was detectable, and the follicle had a diameter of 35 mm.

### 2.3. Cytobrush

The EC method was performed using a Citology Brush (Minitube GmbH Tiefenbach, Germany), following the standard procedure previously described for the mare [12]. Jennies were placed inside a restrained stock, the tail was bandaged and suspended to minimize a potential contamination; the vulva and the perineal region were thoroughly washed with a solution of povidone-iodine (Betadine^®^, MEDA Pharma S.p.A., Milan, Italy) and rinsed with warm water and dried.

The operator equipped with a sterile glove sleeved arm (IMV-Technologies, Franch) allowed the introduction of the double-guarded uterine cytology brush; inside the uterus, the brush was taken out of the protective tube and gently rotated for at least 10 s, alternately to the right and left, near the base’s uterine horn, to obtain cellular endometrium samples; the brush was subsequently re-entered into the protection tube before leaving the uterus. CB samples were immediately set up for cytology evaluation by gently rotating the brush on the glass microscope slide (Frosted slides 26 × 76 mm, cod. VBS654/50, Biosigna, Italy) and then dried in the air.

### 2.4. Citology

The cytological samples were stained with May-Grünwald Giemsa, dried in the air, mounted and examined under an optical microscope at 100×, 400× and 1000× magnification. Only smears of good quality were used to interpretation. All cytological samples were evaluated by board-certified clinical pathologist. First, the smears were evaluated, to appreciate cellular areas at low power (100×); second, to confirm the types of cell present (400×); and, finally to quantify the number of endometrial epithelial cells/×40 high-power fields (HPF). Both raft or clump, monolayer clusters or single endometrial epithelial cells intact, distorted or fragmented (+: low, ++: moderate and +++: high) were recorded. In addition, the cytoplasmic characteristics, such as vacuolation or streaming, and nuclear characteristics and background were also recorded (1000×). Smears were regarded as indicative for inflammation if the amount of PMNs was higher 2% in 200 cells [8].

### 2.5. Statistical Analyses

The descriptive statistics were applied using the Statistica 8.0 for Windows (Statsoft Inc., Bloomberg, NY, USA). The Friedman test was used to compare these rankings as well as the estimated number of endometrial epithelial cells within the most cellular areas of the smears. Cell count data were pooled considering timing of estrus (D-21), early (D-1) and late diestrus (D-14) according to age (younger: 4 to 12 years old, and older: more than 12 years) and if jennies were primiparous and multiparous. Those variables that did not demonstrate a normal distribution (endometrial epithelial cells, macrophages, lymphocytes and eosinophils) were analyzed by post-hoc Dunn multiple comparison test. Was considered a level of significance of *p* < 0.05.

## 3. Results

**Figure 2:**
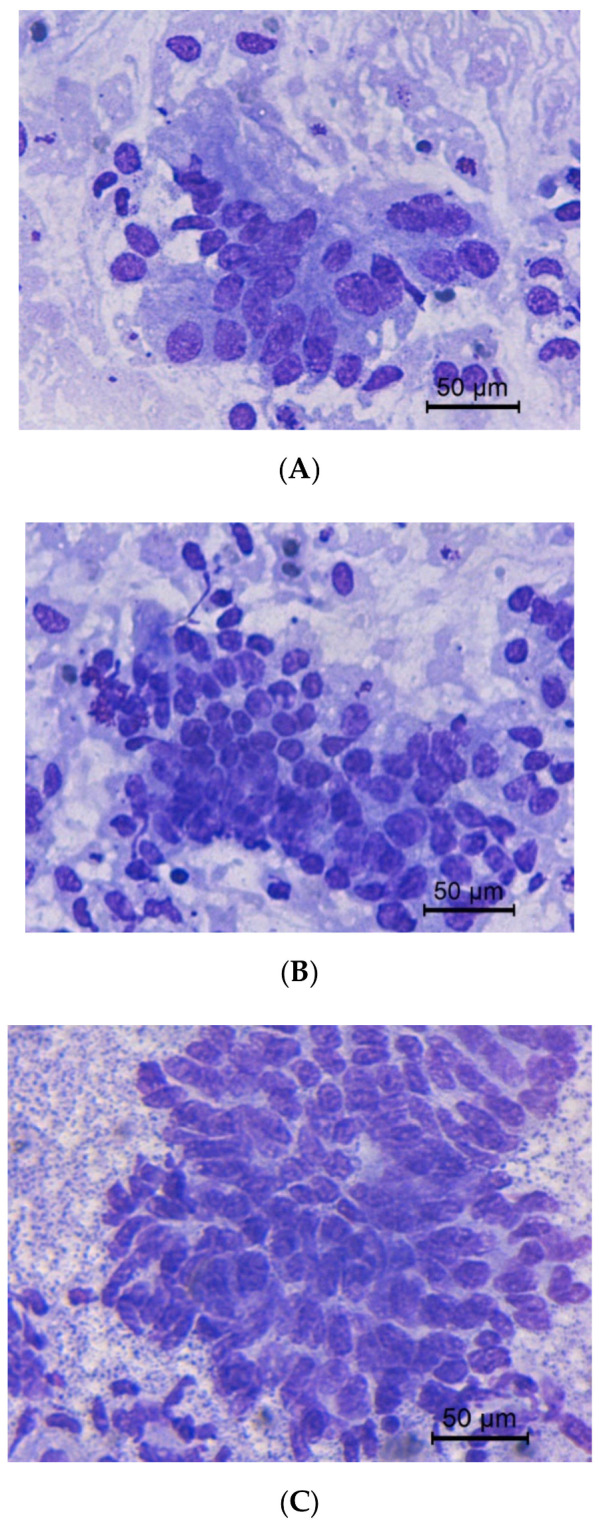
Scores of cellularity in jennie’s cytobrush Examples of low (**A**), moderate (**B**), and high (**C**). The predominant cells are endometrial epithelial cells, seen individually and in clusters. Modified Wright’s stain, 400× magnification.

The quantitative endometrial epithelial and PMNs cells are presented in Table 2. The count of epithelial cells in estrus was significantly higher than early and late diestrus (*p* < 0.05). In addition, in estrus dense and intact exfoliated endometrial epithelial cells were found (*p* < 0.05). Clusters and monolayer distorted cells were more frequently in diestrus than in estrus. Endometrial epithelial cells in estrus presented columnar morphology, becoming more cuboidal during diestrus. Morphological appearance of endometrial epithelial cells in estrus, early and late diestrus is shown in Figure 2.

Compared to young or primiparous females, counts of endometrial epithelial cells and PMNs were higher in older and multiparous jennies (*p* < 0.05). Significant differences in number of PMNs between estrus and late diestrus period were found (Table 3).

## 4. Discussion

The results of this study show significant differences in the number and morphology of endometrial epithelial cells in smears of different phases of estrous cycle in jennies. The number of endometrial epithelial cells in estrus in jennies was similar to the previously obtained by other [8,13]. Fibers from a brush can scrape the endometrial surface and can penetrate deeper, thus enabling collection of a greater number of cells. Consequently, the evaluation of smears obtained from CB provides a higher number of harvested cells, allowing a homogeneous and satisfactory technique, with a more rapid evaluation of the smear.

Compared to the late diestrus, in estrus and early diestrus, different clusters and monolayer dense or elevated number of endometrial epithelial cells were observed. Previous results reported in mares showed major free endometrial epithelial cells during estrus and minor endometrial epithelial cell rafts (including 4 or more cells in closed apposition) during the diestrus [23]; moreover, while the isolated endometrial epithelial cells raft overlap in estrus, this proportion rapidly falls after ovulation [22]. It is known that in the estrogenic phase, cell proliferation and increased uterine vascularization occur; however, during the later ovulation, the proliferation of endometrium decreased to basal values and remained low, while progesterone concentrations remained elevated during diestrus [32,33]. Nevertheless, these results contrast to data obtained by Kozdrowski et al. [4], in which the number of endometrial epithelial cells in smears, obtained by CB, was significantly higher in diestrus compared to estrus.

**Figure 3:**
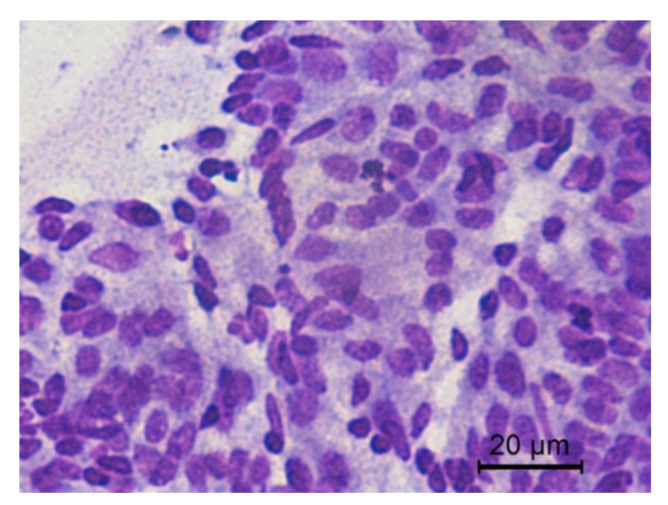
Jenny’s endometrial cytology collected by CB in estrus. Endometrial epithelial columnar cells. Dense and granular background is present. Modified Wright’s stain 400×.

**Figure 4:**
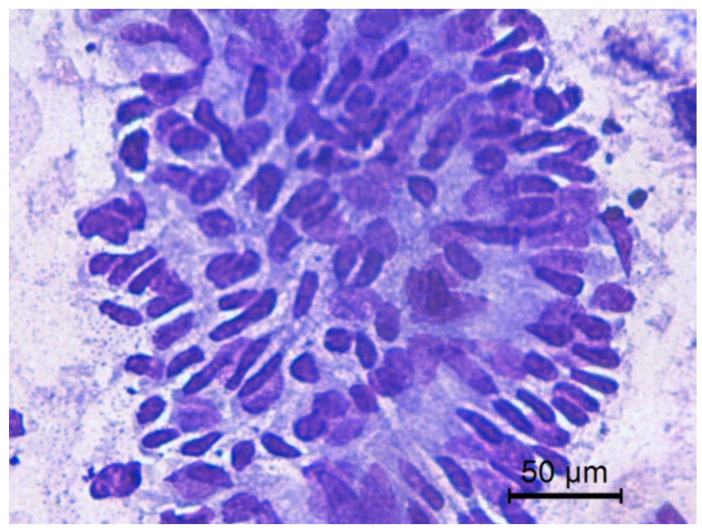
Jenny’s endometrial cytology collected by CB in estrus. Endometrial epithelial columnar cells. Modified Wright’s stain 400×.

**Figure 5:**
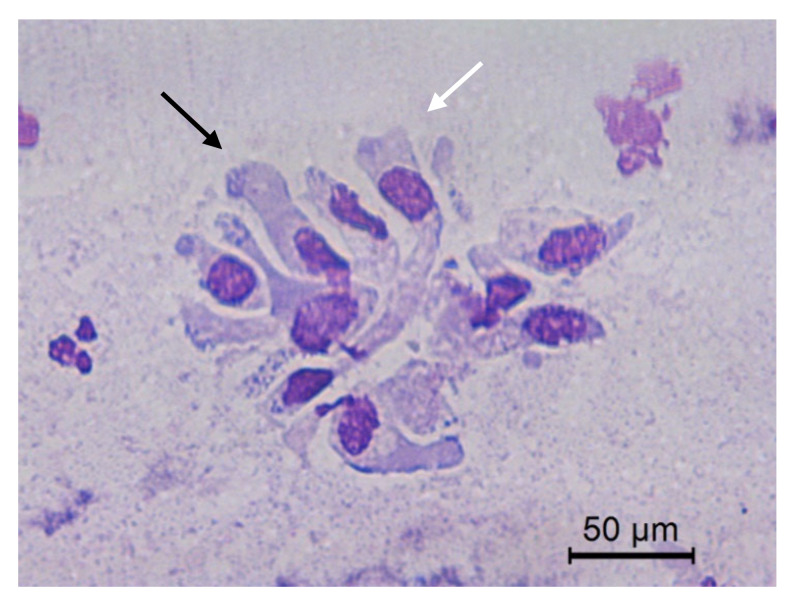
Jenny’s endometrial cytology collected by CB in estrus. Individualized endometrial epithelial cells in apposition, elongated cytoplasm, cilitated (black arrow) and non ciliated (white arrow). Granular background present. Modified Wright’s stain 400×.

**Figure 6:**
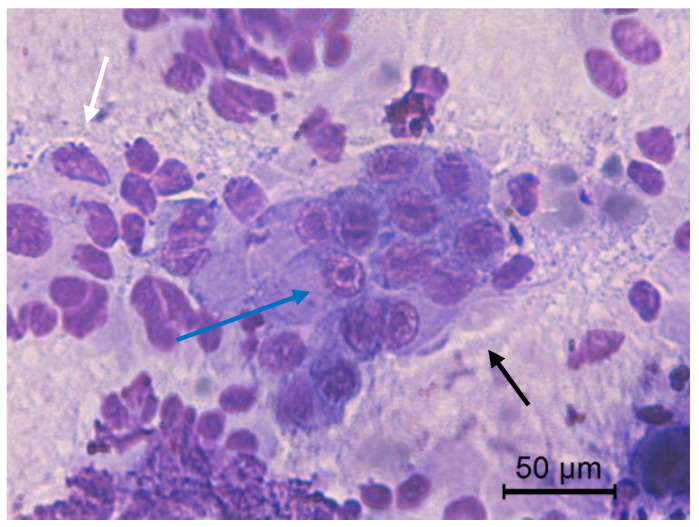
Jenny’s endometrial cytology collected by CB in estrus. Tall (black arrow) and low (white arrow) columnar endometrial epithelial cells. Evident nucleoli (blue arrow). Dense and proteinaceus background present. Modified Wright’s stain 400×.

**Figure 7:**
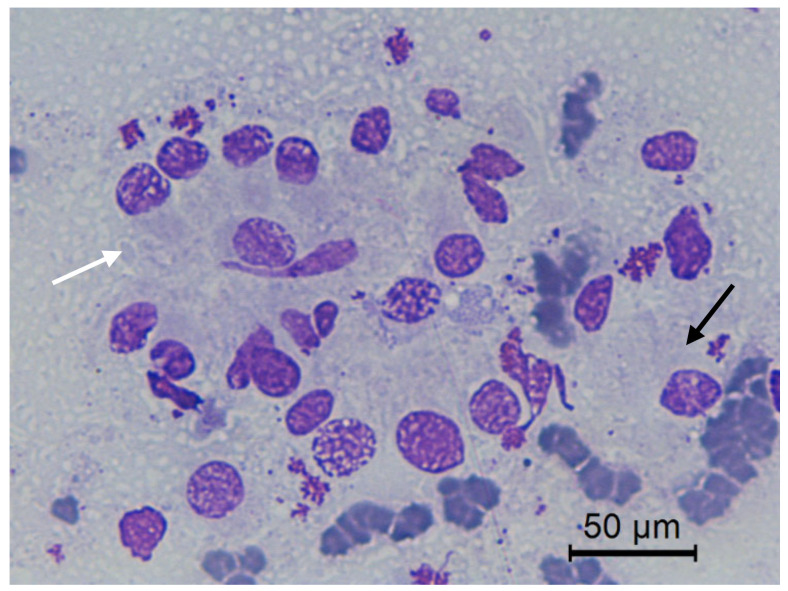
Jenny’s endometrial cytology collected by CB in early diestrus. Cluster (white arrow) and individual (black arrow) endometrial epithelial cells. Granular background present. Modified Wright’s stain 400×.

**Figure 8:**
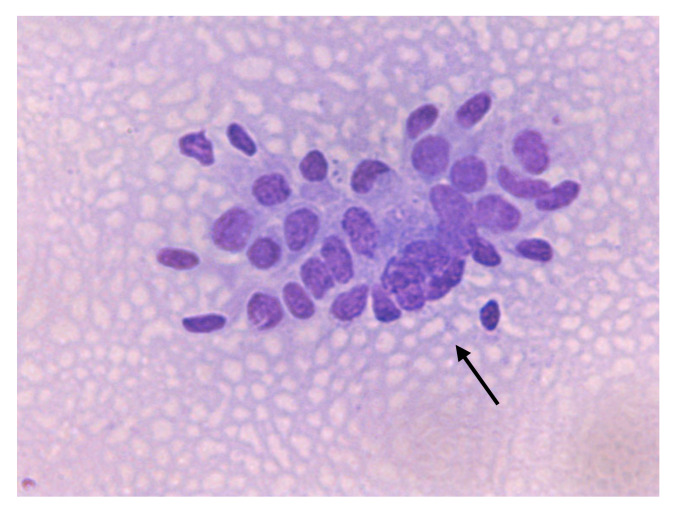
Jenny’s endometrial cytology collected by CB in late diestrus. Cluster, typical enlarged and foamy aspect of endometrial epithelial cells (black arrow). Dense background present. Modified Wright’s stain 400×.

The transition of endometrial epithelial cells from columnar in estrus to cuboid during diestrus in jennies is in agree with data observed in mares [34]. After ovulation, nuclei of endometrial epithelial cells decrease in size, and the cytoplasm is barely visible, becoming more cuboidal during luteal phase, as described in mares [35] (Figure 3). Endometrial epithelial cells in estrus showed relatively large chromatin nuclei eccentrically located and little cytoplasm. Some endometrial epithelial cells had cilia and others not. Ciliated cells were columnar or elliptical and were found in clumps, with eosinophilic cytoplasm and basophilic nucleus located in basal position. Non-ciliated columnar cells or mucus-secreting cells showed an affinity for basic colorant and had a large purple to violet nucleus surrounded by light or light blue cytoplasm. A singular round nucleolus was visible in some cell (Figure 4, Figure 5, Figure 6, Figure 7, Figure 8). The mucous thread pattern, originated in endometrial epithelial cells, was relatively thick and intensely stained, as was reported in normal mares [1].

**Figure 9:**
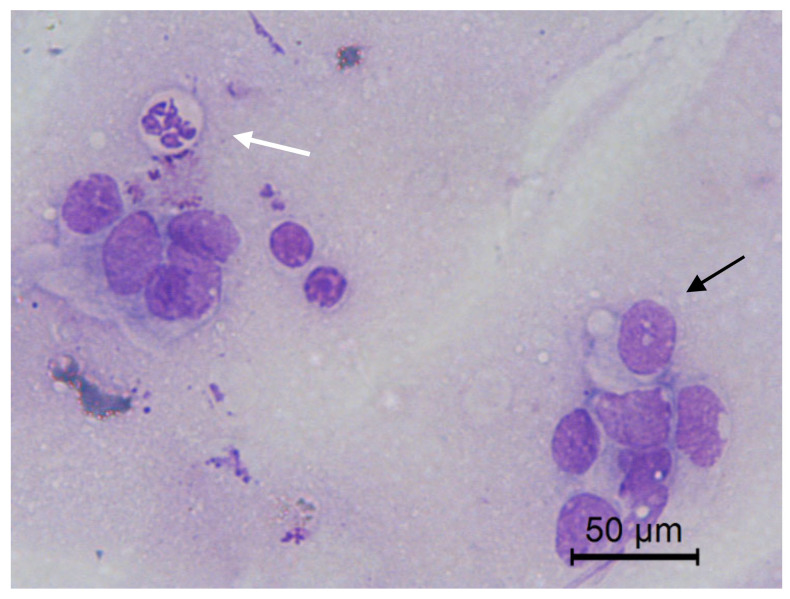
Jenny’s endometrial cytology collected by CB in early diestrus. Cuboidal endometrial cells, vacuolization (black arrow) and neutrophil (white arrow). Dense background present. Modified Wright’s stain 1000×.

Although normal cell clusters were maintained at the start of the diestrus, endometrial epithelial cells, morphologically, showed a more individualized pattern (clusters and monolayer), evident in late diestrus (Figure 8). Furthermore, morphological changes, as distorted or fragmented cells, cytoplasmic vacuolization in some of them and pyknotic nuclei, were found (Figure 9). Similar changes of endometrial epithelial cells during estrus and diestrus in bitches [36] and female cat [37] were documented. However, a high number of altered or degenerated endometrial epithelial cells was also associated with alterations in sample preparation, handling, or storage prior to staining [38].

**Figure 10:**
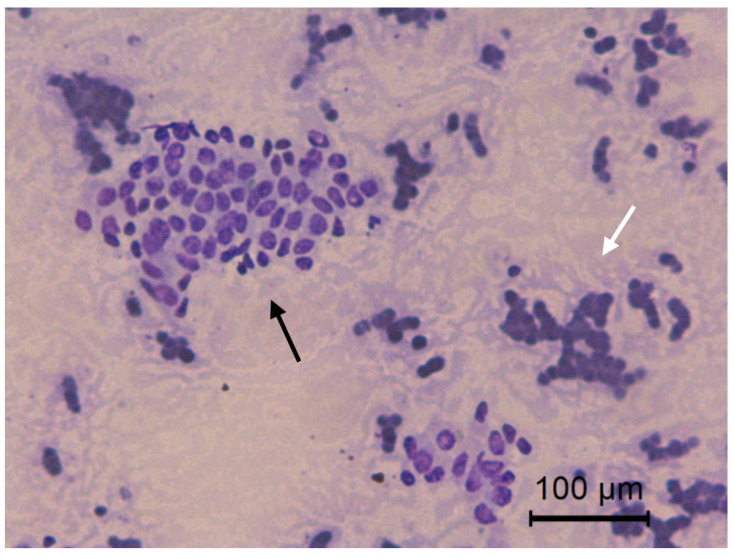
Jenny’s endometrial cytology collected by CB in late diestrus. Cuboidal endometrial cells (black arrow). Dense background and abundant red blood cell (white arrow) present. Modified Wright’s stain 100×.

**Figure 11:**
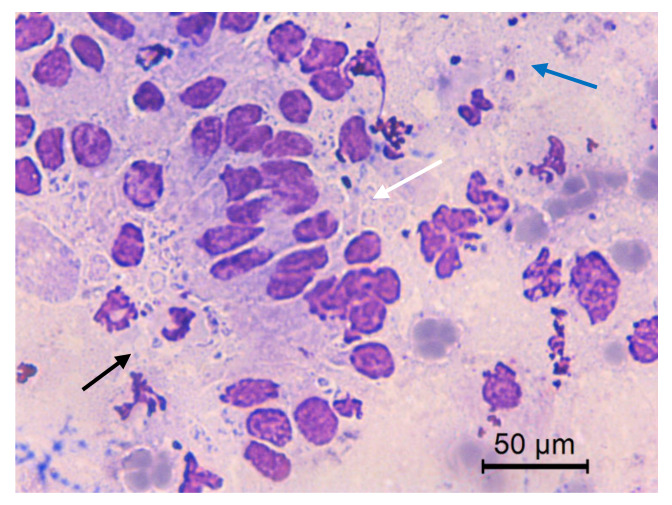
Jenny’s endometrial cytology collected by CB in estrus. Distorted endometrial epithelial cells (white arrow). Clear background, degenerate PMNs (black arrow), RBC, debris and bacteria (blue arrow) are present. Modified Wright’s stain 400×.

This study was performed using the jenny that presumably had a healthy uterus. The absence of inflammatory cells on the EC is considered normal. Despite the expectation that all animals were healthy, a few had slight increased numbers of PMNs in estrus compared to diestrus, as previously observed in healthy mares [4] (Figure 9). Some studies showed that samples obtained in early estrus may not contain inflammatory cells, since PMNs’ migration into the uterine lumen during the period of waning progesterone (P_4_) dominance is lower than during maximal estrogen dominance [2,9]. Nonetheless, the time post-ovulation does not seem to display any effect on EC parameters in mares. Moreover, a small resident amount of PMNs in the endometrium was demonstrated from 24 to 96 h after ovulation, as well as during proestrus, in healthy mares [8,9,39]. In healthy mares, less than 1 to 2 PMNs per five microscopic HPF (×40) during estrus normally exists [21], being necessary to categorize a sample as inflammatory if the greater than five PMNs cells per 10 HPF (×40) are presented [40]. As the percentage of inflammatory cells is normally low and the exfoliation of endometrial epithelial cells is abundantly in estrus in the jenny’s smears, so the ratio of endometrial epithelial cells to PMN less than 40 to one rules out some type of inflammation, as reported in mares. It is unclear whether this is due to unanticipated endometritis or due to a possible contamination of the sample. Note that the disruption of the epithelial lining, due to CB method, which is indicated by the presence of RBC, might also result in a higher number of PMNs in the smear [9,41,42] (Figure 10). The usual inflammatory cells are the PMNs since they are the first to migrate in response to the agent. PMNs in the endometrial luminal compartment are considered by several authors to be the most accurate diagnosis of endometritis in the mare [5,8,39]. Interestingly, a jenny in estrus presented 7–8 PMNs per 10 HPF (400×), but in this case the PMNs were degenerate. Degenerative PMNs signs included changes in the appearance of the cellular membrane, hypersegmentation of the nucleus, vacuolization and cell swelling. In our case cannot assess bacterial type (Figure 11).

However, some samples in estrus of jennies showed the presence of some extracellular bacteria, with a low percentage of PMN (<0.5%). In jennies, the presence of endometrial cells, debris and the finding of occasional or no inflammatory cells were considered signs of a healthy endometrium (Figure 10). However, the number of PMN increases dramatically after insemination or normal mating [12,20,43]. The deposited semen is the main cause of the uterine inflammatory response, which triggers the strong chemotaxis of PMN and the expression of cytokines after insemination [10]. 

In mares elevated PMNs during estrous are rare. Overbeck et al. [44] identified higher value (>2% of all cells were PMNs) related positive culture, no showed in jennies. Some infections, as recent low-grade fungal endometritis and Gram-negative infections (*E. coli* and *P. aeruginosa*) are not always accompanied by an increase in inflammatory cells, increasing the risk of false negative diagnosis [5,44]. Nielsen et al. [43] noted that 50% of the females with a positive culture for *E. Coli* had inflammatory cells in their cytology and that 70% of the mares positive for other microorganisms were associated with the presence of inflammatory cells in the cytological study. Several samples may be needed in some cases, according to the increased sensitivity of endometritis diagnosis by cytology [45].

Advance of age and multiparous condition predispose to infection of the uterus. According to LeBlanc and Causey [45], the mares with minor number of parturitions showed a cervical morphology free of alterations, and those with the highest number of births suffered defects in the physical barriers of the cervix, such as lacerations and adhesions, predisposing the entrance of bacteria into the uterus. Some authors also expressed the importance attributed to the integrity of the anatomical reproductive tract, participating in immune barriers and ensuring a health uterine environment [46,47,48]. On the contrary, older mares have a 25 times higher risk than young mares of endometritis [49,50]. The causes predisposing to endometritis in mares are related to the progressive loss of uterine tone and the decrease in the response of cellular immunity, represented by a lower rate of division of T lymphocytes and cytokines’ production.

**Figure 12:**
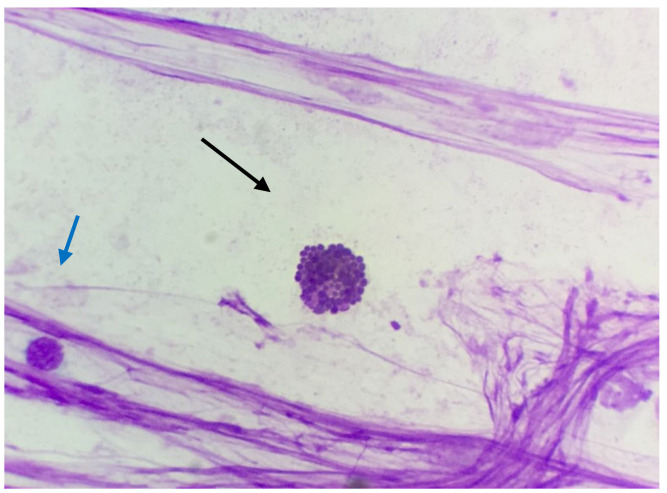
Jenny’s endometrial cytology collected by CB in estrus. Eosinophil (black arrow) and lymphocyte (blue arrow). Proteinaceous background, Modified Wright’s stain 1000×.

The presence of lymphocytes and macrophages could characterize the inflammatory process as chronic [34]. Lymphocytes appear as small, relatively dark cells, with scant cytoplasm [1]. Macrophages are typically large, with a foamy, pale-gray to light-blue cytoplasm, and may have bacteria in the vacuoles of cytoplasm [1] (Figure 12). In a resolving or achronic endometrial inflammatory response, the predominance of eosinophils, lymphocytes and macrophages can be shown [34]. Asinus eosinophil is similar to mare in EC have faintly pink-staining, large, round intracytoplasmic granules and a lobulated nucleus (Figure 12). In cases of pneumovagina, fungal infection, during anaphylactic responses or urine reflux into the uterus, eosinophils can be found in the sample [1,51,52]. Miró and Papas [11] considered that the presence of EOSs is one physiologic finding of endometrium during estrus as well as after inseminationin healthy jennies.

In the same way as in the mare, CB collection is an adequate method to evaluate the endometrial epithelial cells in cycling jennies. The cytological extensions by CB provide higher cellularity [8,9,13], adequately identify the morphology of endometrial epithelial cells, the changes that exist during the estrous cycle, and the presence of inflammatory cells (PMNNs, lymphocytes and eosinophils) [9], although due to their potential for invasiveness, contamination with proteinaceous material or detritus cannot be ignored, as occurs in the mare.

The number and morphology of endometrial epithelial cells considerably change during the estrous cycle. Compared to the diestrus, endometrial epithelial cell exfoliation is significantly more abundant in estrus. Endometrial epithelial cells show columnar morphology during estrus, becoming more cuboidal during diestrus. Degenerative changes observed in later diestrus compared with normal morphology of endometrial epithelial cells in estrus were shown. Although scarce segmented neutrophils are a common response during estrus, degenerate neutrophils are related with endometritis in jennies.

## Figures and Tables

**Table 1:** Cellular types that may be encountered on cytological analysis, their origin and significance in jennies [11,16,17,18,19,20].

Cellular Types	Origin	Significance (Indication of)
Endometrial epithelial cells	Luminal endometrium	Normal
Neutrophils (PMNs)		Acute inflammation
Eosinophils		Pneumouterus
Macrophages		Chronic inflammation or resolving acute inflammation
Lymphocytes		Chronic inflammation
Red blood cell (RBC)		Trauma, inflammation, postpartum

**Table 2:** Endometrial epithelial cellular count (Mean ± SD) in jennies.

Groups	N		Endometrial Epithelial Cells			Background
			Epithelial cells (cells/HPF)	Dense, monolayer and clusters	Intact, distorted or fragmented	Clear, proteinaceous or debris
Cyclic phase	8	Estrous (D-21)	89.28 ± 2.75 ^a^	Dense +++ and clusters +	Intact	Proteinaceous/Clear
	8	Early diestrus (D-1)	79.37 ± 1.99 ^a^	Clusters ++ and monolayer	Intact and distorted	Proteinaceous/Clear
	8	Later diestrus (D-14)	68.88 ± 4.79	Clusters + and monolayer	Distorted and fragmented	Clear/debris
Age	3	Young: 4–12 years	79.75 ± 1.94	Dense and clusters ++	Intact	Proteinaceous/Clear
	5	Older: >12 years	83.23 ± 2.41	Dense and clusters ++	Distorted and fragmented	Clear/Debris
Number of births	3	Primiparous	89.80 ± 1.73 *	Dense +++	Intact	Proteinaceous
	5	Multiparous	83.40 ± 1.73	Clumps +++	Intact and distorted	Clear/Debris

D: day; +: low, ++: moderate and +++: high cluster, dense or clumps. Significant differences to *p* < 0.05. ^a^ D-21 vs D-1 and D-14. * Primiparous vs Multiparous jennies.

**Table 3:** Neutrophil count (Mean ± SD) in jennies.

	N	Groups	PMNs	
			%	Cells/Field
Cyclic phase	8	Estrous (D-21)	0.3	1.15 ± 0.22 ^a^
	8	Early diestrus (D-1)	0.2	0.40 ± 0.11
	8	Later diestrus (D-14)	0.1	0.17 ± 0.04
Age	3	Young: 4–12 years	0.2	0.34 ± 0.10
	5	Older: >12 years	0.3	0.55 ± 0.08
Number of births	3	Primiparous	0.2	0.56 ± 0.13
	5	Multiparous	0.3	1.14 ± 0.13 *

PMN: neutrophil; D: day. ^a,^* Different superscripts indicate significant differences to *p* < 0.05.
